# The association between weight-adjusted-waist index and increased urinary albumin excretion in adults: A population-based study

**DOI:** 10.3389/fnut.2022.941926

**Published:** 2022-08-12

**Authors:** Zheng Qin, Kaixi Chang, Qinbo Yang, Qiao Yu, Ruoxi Liao, Baihai Su

**Affiliations:** ^1^Department of Nephrology, National Clinical Research Center for Geriatrics, Med-X Center for Materials, West China Hospital, Sichuan University, Chengdu, China; ^2^Med+ Biomaterial Institute of West China Hospital/West China School of Medicine of Sichuan University, Chengdu, China

**Keywords:** weight-adjusted-waist index, obesity, albuminuria, NHANES, cross-sectional study

## Abstract

**Aims:**

The negative effect of obesity on kidney health has been reported. The association between weight-adjusted-waist index (WWI, a newly developed adiposity index) and albuminuria has not been reported earlier.

**Methods:**

This cross-sectional study was conducted among adults with complete data about WWI and urinary albumin-to-creatinine ratio (ACR) in 2005–2018 National Health and Nutrition Examination Survey (NHANES). WWI was calculated as waist circumference (WC) divided by the square root of weight. Weighted multivariable logistic regression and generalized additive model were employed to explore the independent relationship between WWI with albuminuria and its non-linearity. A two-piecewise linear regression model was used to calculate the threshold effect. Subgroup analysis and interaction tests were also performed.

**Results:**

A total of 36,921 participants were enrolled with a prevalence of albuminuria of 9.32%. The prevalence of albuminuria increased with the higher WWI tertiles (Tertile 1: 5.31%, Tertile 2: 8.23%, Tertile 3: 15.65%). WWI was positively associated with a higher likelihood of albuminuria (OR = 1.28, 95% CI: 1.15–1.43), and this relationship remains stable in subgroups (all P for trend > 0.05). Non-linear positive relationships were detected in females with a breakpoint of 10.93. A positive association between WWI and albuminuria (OR = 1.39, 95% CI: 1.20–1.61) was observed on the right of the breakpoint, while the association on the left was of no statistical significance. WWI showed a stronger correlation with albuminuria (OR = 1.28) than other markers of obesity including body mass index (BMI, OR = 1.02) and WC (OR = 1.01).

**Conclusion:**

Weight-adjusted-waist index levels were positively related to an increased likelihood of albuminuria in United States adults and showed a stronger relationship than BMI and WC. Our findings indicated that WWI may serve as a simple anthropometric index to predict albuminuria.

## Introduction

Increased urinary albumin excretion is an important indicator for early kidney disease (1). Urinary albumin/creatinine ratio (ACR) of more than 30 mg/g (3.4 mg/mmol) has been recognized as a threshold for abnormal urinary albumin increase. It could be an easily measured marker of diffuse endothelial dysfunction and may indicate underlying macrovascular and microvascular diseases (2, 3). Many epidemiological studies have reported that albuminuria is a robust independent risk factor for cardiovascular events and even all-cause mortality (4). It is also considered to have biological plausibility as a surrogate endpoint for the progression of chronic renal disease (CKD) (5).

Obesity is a global health problem. A population-based study of obesity projections suggested that nearly 1 in 2 adults would have obesity in the United States by 2030 (6). Body mass index (BMI) was a traditional parameter to evaluate obesity; however, it could not distinguish between lean mass and fat mass (7). In recent years, visceral fat has been proposed to more accurately reflect adverse metabolic profiles, which is often associated with abdominal obesity (8). Thus, Park et al. first proposed a new adiposity index termed the weight-adjusted-waist index (WWI), which standardizes waist circumference (WC) for body weight and is easy to measure. Therefore, WWI can draw out the benefits of WC and weaken the correlation with BMI, thus reflecting mainly weight-independent centripetal obesity. Several studies have demonstrated the positive association between WWI with new-onset hypertension, diabetes, and even the all-cause and cardiovascular mortality (9–11). Kim et al. confirmed that WWI could be an indicator of fat and muscle composition changes related to aging commonly applicable to all race/ethnic groups (12).

Obesity has been reported to impair renal function (13–15). Weight loss by medications or surgery also has been proven beneficial for the alleviation of albuminuria and CKD progression (16, 17). However, the relationship between WWI and abnormal urinary albumin has not been reported before.

Thus, this study sought to explore the association between WWI and increased urinary albumin excretion among the general adult in the United States using the data from the National Health and Nutrition Examination Survey (NHANES).

## Materials and methods

### Survey description

Cross-sectional data were obtained from NHANES, a national study to evaluate the nutrition and health status in the United States conducted by the National Center for Health Statistics (NCHS). It was conducted with a complex multistage probability design to obtain a nationally representative group of non-institutionalized United States residents (18). Participants completed an in-home interview to provide demographic, socioeconomic, and health-related information. Physical and laboratory examinations were conducted in a mobile examination center (MEC).

The Research Ethics Review Board of the NCHS approved all NHANES study protocols, and written informed consent was obtained from all survey participants. All detailed NHANES study designs and data are publicly available at www.cdc.gov/nchs/nhanes/. This study followed the Strengthening the Reporting of Observational Studies in Epidemiology (STROBE) reporting guidelines for cross-sectional studies (19).

### Study population

**FIGURE 1:**
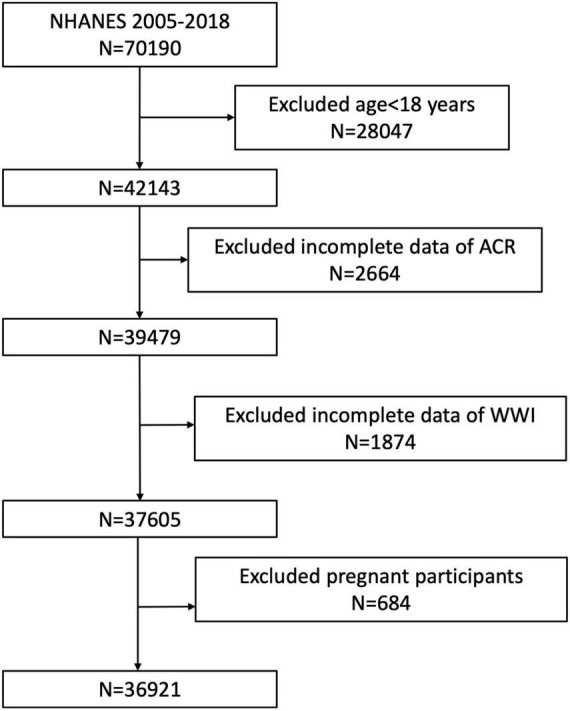
Flowchart of the sample selection from NHANES 2005–2018. A total of 70,190 participants were enrolled at first, and after the exclusion of participants aged < 18 years (*n* = 28,047), missing the data about ACR (*n* = 2,664), WWI (*n* = 1,874), and pregnant (*n* = 684), 36,921 eligible participants were included in our final analysis.

Our study population was recruited from the NHANES 2005–2018. Participants with complete data about ACR and WWI were enrolled in our analysis. A total of 70,190 participants were enrolled at first, and after the exclusion of participants aged < 18 years (*n* = 28,047), missing the data about ACR (*n* = 2,664), WWI (*n* = 1,874), and pregnant (*n* = 684), 36,921 eligible participants were included in our final analysis (Figure 1).

### Assessment of weight-adjusted-waist index

The WWI is an anthropometric index on WC and weight to estimate obesity. A higher WWI score suggested an increased degree of obesity. The body measures data about WC and weight were collected in the MEC by trained health technicians. The WWI for each participant was calculated as WC in centimeters divided by the square root of weight in kilograms and then rounded to two decimal places. We treated WWI as a continuous variable in the analysis, and participants were grouped based on the WWI tertiles for further analysis. WWI was designed as an exposure variable in our study.

### Assessment of increased urinary albumin excretion

Urine samples of NHANES participants were obtained in MEC. Urinary albumin and creatinine were determined by a solid-phase fluorescent immunoassay and modified Jaffe kinetic methods. ACR was calculated by dividing the urinary albumin concentration by the urinary creatinine concentration. According to previous studies, increased urinary albumin excretion (albuminuria) was defined as ACR > 30 mg/g (20, 21). In our analysis, albuminuria was designed as an outcome variable.

### Assessment of covariates of interest

Demographic covariates in our study included gender (male/female), age (years), race (Mexican American/other Hispanic/non-Hispanic White/non-Hispanic Black/other races), education level (less than high school/high school or general educational development/above high school/others), and smoking status (never/former/current/unknown). Several anthropometric and laboratory covariates also have been included, such as body mass index (BMI, kg/m^2^, calculated as weight in kilograms divided by height in meters squared), systolic blood pressure (SBP, mmHg), diastolic blood pressure (DBP, mmHg), fasting plasma glucose (μIU/ml), hemoglobin A1c (%), serum creatinine (mg/dl), serum uric acid (μmol/L), total cholesterol (mmol/L), high-density lipoprotein cholesterol (HDL-C, mmol/L), low-density lipoprotein cholesterol (LDL-C, mmol/L), alanine transaminase (ALT, U/L); aspartate transaminase (AST, U/L), and triglycerides (mmol/L). The health condition variates composed of hypertension (yes/no) and diabetes (yes/no) were also included. Diabetes was defined as taking hypoglycemic medications or ever been told by a doctor that have a diagnosis of diabetes, a hemoglobin A1c level ≥ 6.5%, a fasting plasma glucose ≥ 126 mg/dl, or a 2-h plasma glucose ≥ 200 mg/dl (22). Hypertension was defined as taking antihypertensive medications, ever been told by a doctor that have a diagnosis of hypertension or having three consecutive systolic blood pressure measurements ≥ 140 mmHg or diastolic blood pressure ≥ 90 mmHg (23). BMI was categorized as < 25, 25–29.9, and ≥ 30 kg/m^2^, which corresponded to normal weight, overweight, and obese. All detailed measurement processes of these variables are publicly available at www.cdc.gov/nchs/nhanes/.

### Statistical analysis

All statistical analyses were conducted according to the CDC guidelines using appropriate NHANES sampling weights and accounted for complex multistage cluster surveys.

In descriptive analyses, either a weighted Student’s *t*-test (for continuous variables) or weighted Chi-square test (for categorical variables) was employed to evaluate the differences among participants grouped by WWI tertiles. Continuous variables are summarized as the means with standard error (SE), and categorical parameters are presented as proportions. To examine the association between WWI and albuminuria, multivariable regression models that accounted for NHANES complex sampling design (sampling weights) were employed in three different models. In Model 1, no covariates were adjusted. Model 2 was adjusted for gender, age, and race. Model 3 was adjusted for gender, age, race, education level, ALT, AST, total cholesterol, serum creatinine, triglycerides, serum uric acid, BMI, SBP, DBP, fasting plasma glucose, hemoglobin A1c (%), HDL-C, LDL-C, hypertension, diabetes, and smoking status. In sensitivity analysis, WWI was converted from a continuous variable to a categorical variable (tertiles) to evaluate its robustness. We also used a generalized additive model (GAM) and smooth curve fittings to address the non-linearity of WWI with albuminuria and each stratification. If a non-linear correlation was observed, a two-piecewise linear regression model (segmented regression model) was used to fit each interval and calculate the threshold effect. Log-likelihood ratio test comparing a one-line model (non-segmented) with a two-piecewise linear regression model was conducted to determine whether a threshold exists. The breakpoint (K) that connects the segments was based on the model that gives maximum likelihood, and it was determined using the two-step recursive method. Subgroup analysis was conducted using stratified multivariable logistic regression models with stratified factors including gender (male/female), age (< 39/40–59/ ≥ 60 years), BMI (normal weight/overweight/obesity), hypertension (yes/no), and diabetes (yes/no). In addition, these stratified factors were also treated as prespecified potential effect modifiers, and an interaction term was added using the likelihood ratio test to evaluate the heterogeneity of associations across subgroups as well. Similarly, we also utilized the smooth curve fittings to detect the non-linearity of WC and BMI on albuminuria, and a two-piecewise linear regression model was employed to further explore their threshold effects. Missing values were input by the median for continuous variables or mode for categorical variables of existing cases of those variables. All analyses were performed using R version 4.1.3 (The R Foundation^1^) and Empower software (X&Y solutions, Inc., Boston, MA, United States^2^). A two-sided *P* < 0.05 was considered statistically significant.

## Results

### Baseline characteristics of participants

A total of 36,921 participants with an average age of 46.34 ± 0.23 years were enrolled, of whom 49.14% were male and 50.86% were female. WWI tertiles 1–3 ranges were 7.72–10.63, 10.63–11.39, and 11.39–15.70; 9.32% of participants were categorized as albuminuria overall, and the prevalence of albuminuria increased with higher WWI tertile (Tertile 1: 5.31%; Tertile 2: 8.23%; Tertile 3: 15.65%; *P* < 0.0001) (Table 1).

**TABLE 1:** Baseline characteristics of study population according to weight-adjusted-waist index tertiles.

Weight-adjusted-waist index	Overall	Tertile 1 (7.72–10.63)	Tertile 2 (10.63–11.39)	Tertile 3 (11.39–15.70)	P for trend
		*N* = 12307	*N* = 12307	*N* = 12307	
Age (year)	46.34 ± 0.23	37.10 ± 0.26	47.88 ± 0.23	56.17 ± 0.26	< 0.0001
**Gender,% (SE)**					
Male	49.14 (0.26)	57.16 (0.54)	50.87 (0.52)	37.02 (0.59)	< 0.0001
Female	50.86 (0.26)	42.84 (0.54)	49.13 (0.52)	62.98 (0.59)	
**Race,% (SE)**					
Mexican American	8.68 (0.67)	6.11 (0.48)	9.84 (0.73)	10.54 (0.94)	0.0006
Other Hispanic	5.65 (0.43)	5.00 (0.41)	6.15 (0.52)	5.87 (0.47)	
Non-Hispanic White	66.74 (1.28)	66.79 (1.24)	65.81 (1.42)	67.76 (1.43)	
Non-Hispanic Black	11.20 (0.68)	14.00 (0.83)	9.83 (0.65)	9.29 (0.66)	
Other Races	7.73 (0.39)	8.09 (0.43)	8.36 (0.50)	6.53 (0.43)	
**Education level,% (SE)**					
Less than high school	16.42 (0.54)	12.33 (0.51)	16.26 (0.67)	21.75 (0.68)	< 0.0001
High school or GED	23.74 (0.47)	21.10 (0.70)	23.92 (0.63)	26.87 (0.67)	
Above high school	59.79 (0.82)	66.53 (1.00)	59.75 (0.99)	51.34 (0.91)	
Others	0.05 (0.01)	0.05 (0.02)	0.06 (0.03)	0.05 (0.01)	
**Smoking status,% (SE)**					
Never	54.38 (0.54)	58.39 (0.75)	52.87 (0.73)	51.10 (0.71)	< 0.0001
Former	23.83 (0.41)	16.55 (0.51)	26.40 (0.57)	29.98 (0.61)	
Current	19.88 (0.43)	21.09 (0.62)	19.86 (0.56)	18.40 (0.56)	
Unknown	1.91 (0.10)	3.98 (0.25)	0.87 (0.08)	0.52 (0.07)	
BMI (Kg/m^2^)	28.82 ± 0.08	25.13 ± 0.07	29.12 ± 0.07	33.13 ± 0.12	< 0.0001
SBP (mmHg)	122.32 ± 0.18	117.25 ± 0.20	122.50 ± 0.24	128.63 ± 0.27	< 0.0001
DBP (mmHg)	70.69 ± 0.18	69.65 ± 0.21	71.88 ± 0.21	70.62 ± 0.23	< 0.0001
Diabetes,% (SE)	8.99 (0.21)	2.22 (0.17)	7.39 (0.31)	19.40 (0.42)	< 0.0001
Hypertension,% (SE)	30.58 (0.47)	14.71 (0.46)	31.45 (0.63)	49.55 (0.59)	< 0.0001
Fasting plasma glucose (μIU/mL)	5.90 ± 0.02	5.45 ± 0.02	5.89 ± 0.03	6.48 ± 0.04	< 0.0001
Hemoglobin A1c (%)	5.60 ± 0.01	5.31 ± 0.01	5.59 ± 0.01	5.96 ± 0.01	< 0.0001
Serum creatinine (μmol/L)	78.22 ± 0.21	79.08 ± 0.25	77.38 ± 0.31	78.12 ± 0.37	0.0060
Serum uric acid (μmol/L)	322.22 ± 0.74	308.95 ± 1.07	323.95 ± 1.09	336.86 ± 1.04	< 0.0001
Total cholesterol (mmol/L)	5.01 ± 0.01	4.85 ± 0.01	5.13 ± 0.02	5.07 ± 0.02	< 0.0001
HDL-C (mmol/L)	1.38 ± 0.01	1.47 ± 0.01	1.35 ± 0.01	1.31 ± 0.01	< 0.0001
LDL-C (mmol/L)	2.93 ± 0.01	2.81 ± 0.01	3.04 ± 0.02	2.97 ± 0.02	< 0.0001
ALT (U/L)	25.27 ± 0.14	23.36 ± 0.19	26.80 ± 0.26	25.85 ± 0.24	< 0.0001
AST (U/L)	25.24 ± 0.10	24.73 ± 0.14	25.63 ± 0.20	25.42 ± 0.20	0.0026
Triglycerides (mmol/L)	1.71 ± 0.01	1.37 ± 0.02	1.83 ± 0.02	2.01 ± 0.02	< 0.0001
Waist circumference (cm)	98.63 ± 0.22	86.61 ± 0.17	99.88 ± 0.15	112.30 ± 0.24	< 0.0001
Weight (kg)	82.29 ± 0.23	74.87 ± 0.25	83.61 ± 0.25	90.08 ± 0.39	< 0.0001
Albumin, urine (mg/L)	32.76 ± 1.30	19.52 ± 1.07	30.23 ± 2.54	52.37 ± 2.42	< 0.0001
Creatinine, urine (mg/dL)	122.93 ± 0.83	130.21 ± 1.42	121.92 ± 1.14	114.94 ± 0.97	< 0.0001
ACR (mg/g)	32.81 ± 1.72	19.17 ± 3.22	28.20 ± 2.32	55.36 ± 2.73	< 0.0001
Albuminuria,% (SE)	9.32 (0.22)	5.31 (0.25)	8.23 (0.35)	15.65 (0.44)	< 0.0001

GED, general educational development; BMI, body mass index; SBP, systolic blood pressure; DBP, diastolic blood pressure; HDL-C, high-density lipoprotein cholesterol; LDL-C, low-density lipoprotein cholesterol; ALT, alanine transaminase; AST, aspartate transaminase; ACR, albumin: creatinine ratio.

### The association between weight-adjusted-waist index and albuminuria

Table 2 shows the association between WWI and albuminuria. Our results demonstrated that higher WWI was associated with an elevated likelihood of albuminuria. A positive association between WWI and albuminuria was detected both in the crude model and minimally/fully adjusted model. After full adjustment, subjects with a unit higher WWI had a 28% increased risk of albuminuria (Model 3: OR = 1.28, 95% CI: 1.15–1.43). This association remained statistically significant after WWI categorized as tertiles. Participants in the highest WWI tertile had a significantly 42% increased risk than those in the lowest WWI tertile (OR = 1.42, 95% CI: 1.11–1.80; P for trend = 0.0052) (Table 2).

**TABLE 2:** Association between weight-adjusted-waist index and albuminuria.

	OR^1^ (95% CI^2^), *P*-value		
	
	Crude model (Model 1)^3^	Minimally adjusted model (Model 2)^4^	Fully adjusted model (Model 3)^5^
Continuous	1.83 (1.74, 1.93), < 0.0001	1.49 (1.40, 1.58), < 0.0001	1.28 (1.15, 1.43), < 0.0001
**Categories**			

**Tertile 1**	**Reference**	**Reference**	**Reference**

Tertile 2	1.60 (1.39, 1.84), < 0.0001	1.23 (1.07, 1.42), 0.0055	1.11 (0.87, 1.42), 0.4086
Tertile 3	3.31 (2.95, 3.71), < 0.0001	2.10 (1.85, 2.38), < 0.0001	1.42 (1.11, 1.80), 0.0052
P for trend	< 0.0001	< 0.0001	< 0.0001

Insensitivity analysis, the visceral adiposity index was converted from a continuous variable to a categorical variable (tertiles).

^1^95% CI: 95% confidence interval.

^2^OR: Odds ratio.

^3^Model 1: No covariates were adjusted.

^4^Model 2: Adjusted for gender, age, and race.

^5^Model 3: Adjusted for gender, age, race, education level, ALT, AST, total cholesterol, serum creatinine, triglycerides, serum uric acid, body mass index, systolic blood pressure, diastolic blood pressure, fasting plasma glucose, hemoglobin A1c, HDL-C, LDL-C, hypertension, diabetes, and smoking status.

Gender, race, SBP, diabetes, hypertension, hemoglobin A1c, serum creatinine, serum uric acid, total cholesterol, and LDL-C remained significantly associated with the odds of albuminuria in the fully adjusted model (Table 3). Compared with male participants, female participants had a 52% increased risk of albuminuria (OR = 1.52, 95% CI: 1.23–1.87). Compared with Mexican American, other Hispanic, non-Hispanic White, non-Hispanic Black, and other races showed a 27, 36, 27, and 26% lower likelihood of albuminuria, respectively. The odds of albuminuria were lowered 38 and 25% in the non-diabetes and non-hypertension population compared with their counterparts. Per unit increase in hemoglobin A1c, serum creatinine, serum uric acid, total cholesterol, SBP, and LDL-C, the odds of albuminuria were elevated by 30%, 1.0%, 0.12%, 74%, 2.1%, and lowered by 50%, respectively (Table 3).

**TABLE 3:** Multivariate logistic regression models of albuminuria.

Variables	OR (95% CI)	*P*-value
Weight-adjusted-waist index	1.28 (1.15, 1.43)	< 0.0001
Age (year)	1.001 (0.995, 1.008)	0.7135
Female (versus male)	1.52 (1.24, 1.87)	0.0001
**Race (versus Mexican American)**		
Other Hispanic	0.73 (0.55, 0.95)	0.0225
Non-Hispanic White	0.64 (0.53, 0.77)	< 0.0001
Non-Hispanic Black	0.73 (0.59, 0.90)	0.0040
Other Races	0.74 (0.57, 0.96)	0.0226
**Education level (versus less than high school)**		
High school or GED	0.91 (0.76, 1.09)	0.2857
Above high school	0.73 (0.58, 0.92)	0.0077
Others	0.78 (0.20, 3.10)	0.7200
**Smoke (versus never)**		
Former	1.08 (0.91, 1.30)	0.3631
Current	1.25 (0.99, 1.57)	0.0587
Unknown	1.52 (0.87, 2.64)	0.1404
BMI (Kg/m^2^)	0.99 (0.98, 1.01)	0.2942
SBP (mmHg)	1.021 (1.016, 1.026)	< 0.0001
DBP (mmHg)	1.003 (0.997, 1.008)	0.3823
Diabetes (no versus yes)	0.62 (0.50, 0.79)	0.0001
Hypertension (no versus yes)	0.75 (0.64, 0.86)	0.0001
Fasting plasma glucose (μIU/mL)	1.01 (0.96, 1.07)	0.6957
Hemoglobin A1c (%)	1.30 (1.17, 1.44)	< 0.0001
Serum creatinine (μmol/L)	1.01 (1.01, 1.01)	< 0.0001
Serum uric acid (μmol/L)	1.0012 (1.0002, 1.0023)	0.0199
Total cholesterol (mmol/L)	1.74 (1.16, 2.61)	0.0078
HDL-C (mmol/L)	0.72 (0.47, 1.11)	0.1312
LDL-C (mmol/L)	0.50 (0.33, 0.75)	0.0013
ALT (U/L)	0.99 (0.98, 1.00)	0.0485
AST (U/L)	1.01 (1.00, 1.02)	0.0534
Triglycerides (mmol/L)	0.84 (0.69, 1.02)	0.0787

The unit for continuous variables and the reference group for categorical variables are provided next to the variables. The OR of albuminuria was each unit increase of continuous variables and compared with the reference group for categorical variables.

**FIGURE 2:**
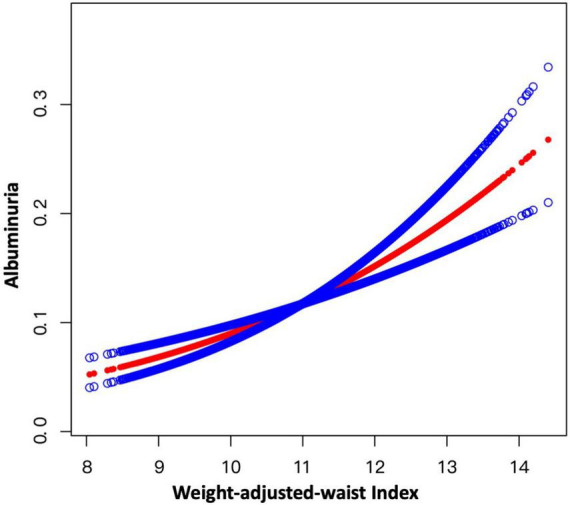
Smooth curve fitting for WWI and albuminuria. A linear relationship between WWI and albuminuria was detected by the generalized additive model.

In addition, the results of smooth curve fitting indicated that there was no non-linear relationship between WWI and the risk of albuminuria in total samples (Figure 2).

### Subgroup analysis

**FIGURE 3:**
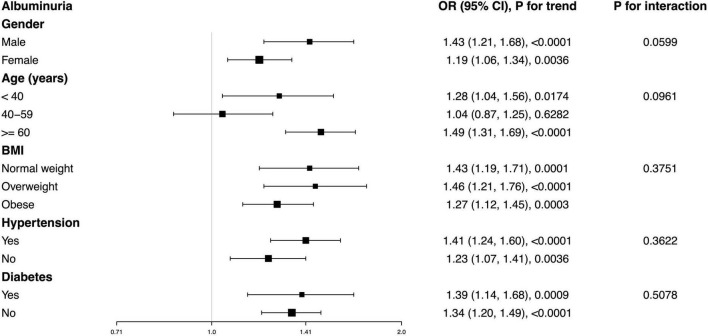
Subgroup analysis for the association between WWI and albuminuria. None of the stratifications including gender, age, BMI, hypertension, and diabetes status significantly affected the positive association of WWI and albuminuria.

Subgroup analysis was conducted to evaluate whether the relationship between WWI and albuminuria was stable among different population settings. Our results indicated that there was no dependence for the association between WWI and albuminuria. As shown in Figure 3, none of the stratifications, including gender, age, BMI, hypertension, and diabetes status, significantly affected the positive association between WWI and albuminuria (all P for interaction < 0.05). The positive association was robust among different subgroups. For example, we detected each unit increase in WWI that corresponded with a 39% greater likelihood of albuminuria in diabetes, and this relationship remained significant in non-diabetes (OR = 1.34, 95% CI: 1.20–1.49).

### Non-linear positive association of weight-adjusted-waist index and albuminuria in females

**FIGURE 4:**
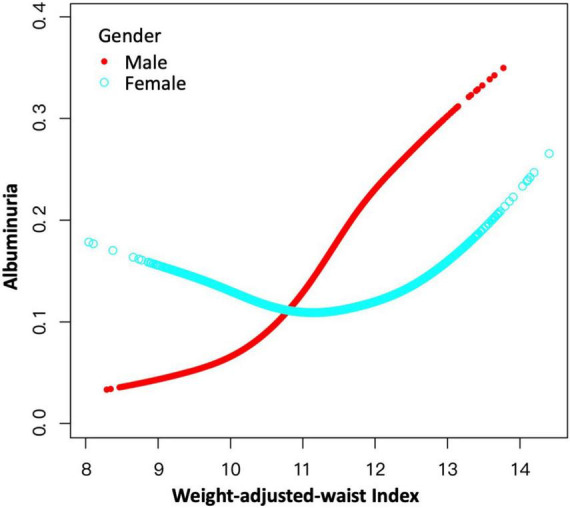
A non-linear positive relationship between WWI and albuminuria in females by the generalized additive model. Non-linear positive relationships were detected in females with a breakpoint of 10.92. WWI positively associated with the likelihood of albuminuria on the right side of the breakpoint, while the association on the left of the breakpoint was of no statistical significance.

We further employed GAM and smooth curve fittings to address the non-linearity for each stratification. There was no non-linear relationship between WWI and albuminuria stratified by age, BMI, hypertension, and diabetes, while the resultant curve of gender exhibited a non-linear relationship in female participants (Figure 4). By the two-piecewise linear regression model, we calculated that the breakpoint (K) was 10.93 for females. On the right of the breakpoint, a positive association between WWI and albuminuria was observed (OR = 1.39, 95% CI: 1.20–1.61). However, no relationship with statistical significance was observed on the left of the breakpoint (OR = 0.79, 95% CI: 0.61–1.02). For male participants, no non-linear relationship has been detected (logarithmic likelihood ratio test *P*-value = 0.070) (Table 4).

**TABLE 4:** Threshold effect analysis of WWI on albuminuria using a two-piecewise linear regression model.

	Male	Female
**Fitting by standard linear model**		
OR^1^ (95% CI^2^)	1.43 (1.22, 1.68)	1.19 (1.06, 1.34)
*P*-value	< 0.0001	0.0036
**Fitting by two-piecewise linear model**		
Breakpoint (K)	9.88	10.93
OR1 (< 11.11)	0.69 (0.32, 1.50) 0.3529	0.79 (0.61, 1.02) 0.0726
OR2 (> 11.11)	1.49 (1.26, 1.76) < 0.0001	1.39 (1.20, 1.61) < 0.0001
OR2/OR1	2.15 (0.96, 4.79) 0.0624	1.75 (1.27, 2.41) 0.0006
Logarithmic likelihood ratio test *P*-value	0.077	< 0.001

Age, race, education level, body mass index, serum creatinine, serum uric acid, SBP, DBP, total cholesterol, triglycerides, fasting plasma glucose, hemoglobin A1c, HDL-C, LDL-C, ALT, AST, smoking, hypertension, and diabetes status were adjusted.

^1^OR: Odds ratio.

^2^95% CI: 95% confidence interval.

### Weight-adjusted-waist index showed a stronger correlation than body mass index and waist circumference for albuminuria

**FIGURE 5:**
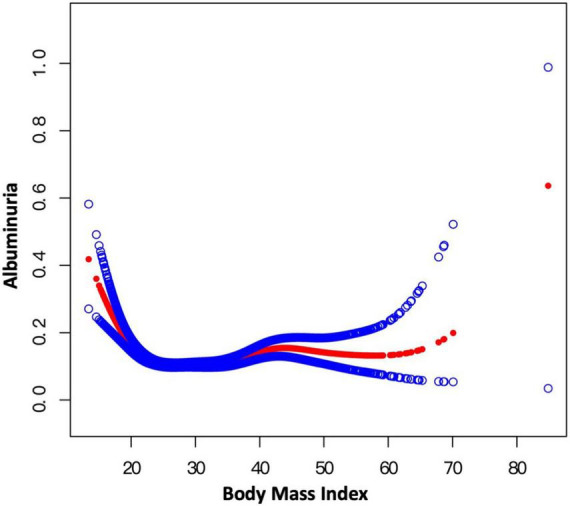
Smooth curve fitting for BMI and albuminuria. Non-linear relationship between BMI and albuminuria was detected by the generalized additive model.

**FIGURE 6:**
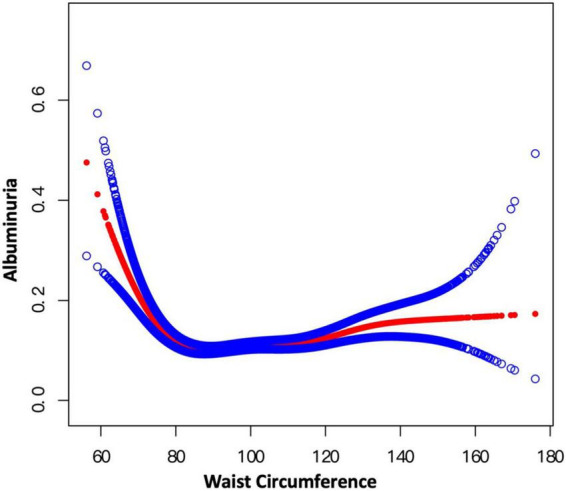
Smooth curve fitting for WC and albuminuria. Non-linear relationship between WC and albuminuria was detected by the generalized additive model.

Smooth curve fitting indicated the non-linearity for the association of BMI with albuminuria (Figure 5) and WC with albuminuria (Figure 6). Then, the segmented regression model was utilized to fit each interval and calculate the threshold effect (Table 5). For the BMI and albuminuria, we detected a breakpoint of 22.57 kg/m^2^. A negative association (OR = 0.80, 95% CI: 0.75–0.84) was observed when BMI was less than 22.57 kg/m^2^, while a positive association (OR = 1.02, 95% CI: 1.01–1.03) was observed when BMI was higher than this point. Similarly, the breakpoint of WC for albuminuria was 80.30 cm. WC was negatively associated with the likelihood of albuminuria on the left of the breakpoint (OR = 0.91, 95% CI: 0.88–0.93), while the association on the left was positive (OR = 1.01, 95% CI: 1.00–1.01). Compared with BMI and WC on the right of their breakpoints, WWI showed a much stronger correlation with the risk of albuminuria (WWI: OR = 1.28; BMI: OR = 1.02; WC: OR = 1.01), indicating that WWI might be a better predictor of the likelihood of albuminuria than other markers of obesity such as BMI and WC.

**TABLE 5:** Threshold effect analysis of BMI and WC on albuminuria using a two-piecewise linear regression model.

	BMI	WC
**Fitting by standard linear model**		
OR^1^ (95% CI^2^),	1.00 (0.99, 1.01)	11.00 (1.00, 1.01)
*P*-value	0.4177	0.1674
**Fitting by two-piecewise linear model**		
Breakpoint (K)	22.57	80.30
OR1 (< 11.11)	0.80 (0.75, 0.84) < 0.0001	0.91 (0.88, 0.93) < 0.0001
OR2 (> 11.11)	1.02 (1.01, 1.03) 0.0007	1.01 (1.00, 1.01) 0.0001
OR2/OR1	1.28 (1.20, 1.36) < 0.0001	1.11 (1.08, 1.14) < 0.0001
Logarithmic likelihood ratio test *P*-value	< 0.001	< 0.001

BMI, body mass index; WC, waist circumference. Age, race, education level, body mass index, serum creatinine, serum uric acid, SBP, DBP, total cholesterol, triglycerides, fasting plasma glucose, hemoglobin A1c, HDL-C, LDL-C, ALT, AST, smoking, hypertension, and diabetes status were adjusted.

^1^OR: Odds ratio.

^2^95% CI: 95% confidence interval.

## Discussion

This study was to evaluate the relationship between WWI and albuminuria among the United States non-institutionalized civilians. In our cross-sectional study with 36,927 participants enrolled, we observed that participants with higher WWI showed an increased likelihood of albuminuria. Subgroup analysis and interaction test showed that this association was similar across different population settings. Non-linear positive relationships were detected in females, and the different correlations of WWI on albuminuria were found on the left and right sides of the breakpoint (WWI = 10.92). In females, WWI was positively associated with the likelihood of albuminuria on the right side of the breakpoint, while the association on the left of the breakpoint was of no statistical significance. We also observed a stronger relationship between WWI and albuminuria than BMI and WC, suggesting that WWI might be a better predictor for albuminuria than other markers of obesity. Our findings indicated that WWI may predict the incidence of albuminuria, and the management of obesity evaluated by WWI may alleviate the abnormal urinary albumin excretion.

To the best of our knowledge, this is the first cross-sectional human study assessing the association between WWI and albuminuria. It has been reported that WWI is a newly developed obesity index that has been explored in various fields, particularly related to cardiovascular diseases (9, 10, 12). We confirmed that WWI had a strong correlation with albuminuria, and the linear positive correlation we found between WWI and albuminuria remained stable in the fully adjusted model. In multivariate logistic regression models, the effects of hypertension, diabetes, and dyslipidemia (i.e., total cholesterol and LDL-C) are the most notable, which reminds us to pay attention to cardiometabolic abnormalities (including high fasting glucose, low high-density lipoprotein cholesterol, and high triglyceride levels and hypertension) (24). Accordingly, these population groups deserve high priority in renal healthcare, as established in landmark randomized trials and recommended in clinical guidelines (25, 26). The same risk factors, namely, study variables including abdominal obesity, significantly increased the risk of microalbuminuria in 1,557 patients with diabetes (27). In subgroup analysis, similar trends were observed among some common sub-populations, and interaction terms for gender, age, BMI, hypertension, and diabetes status with albuminuria were not significant in any of the stratifications, indicating that higher WWI predisposes general populations to albuminuria. We found that female participants had a 52% greater risk of albuminuria than male participants, and the approximately same number of male and female participants helped to exclude the errors due to the differences of sample size. Several biological factors are involved in the pathophysiology of albuminuria, and of these, sex hormones directly and indirectly are thought to regulate renal hemodynamics, oxidative stress, and inflammation (28). However, muscle mass and urinary creatinine excretion are usually lower in women than in men (29, 30), which may lead to an overestimation of albuminuria in women when using urinary ACR, a preferable test recommended by guidelines for quantifying albuminuria (31, 32).

Additionally, the correlation between WWI and albuminuria also appeared to be more sex specific. We found a linear relationship between WWI and albuminuria in male and a non-linearity in female, which may be largely due to the specific fat distribution. For instance, the fat located as visceral fat depot is typically observed in males, while it is more frequently found in the hips of females (33). Similar gender differences were also observed in several studies (34, 35). For the non-linear correlation observed in the female groups, we further concluded that the inflection point of WWI was 10.93, which means that the correlation between WWI and albuminuria is meaningful when the WWI of women reaches this threshold, which was similar to the obesity thresholds used in other studies (9, 36). In addition, Mexican Americans were considered to have a higher likelihood of albuminuria, which is consistent with previous findings suggesting the role of genetic and unmeasured sociocultural factors (37, 38).

Many indicators have been used to evaluate obesity, especially targeting the recognized harmful intra-abdominal fat mass. BMI was the most widely used anthropometric measurement, while it cannot distinguish lean mass and fat mass. WC was proposed as an alternative measure for the indirect evaluation of increased visceral fat, also being an indicator of abdominal obesity for diagnosing metabolic syndrome (MetS). The Look AHEAD Study analyzing 1,351 participants suggested that the highest quartile of WC (OR = 1.75, 95% CI: 1.42–2.15) was significantly associated with albuminuria compared with the lowest quartile (13). However, similar to BMI, WC alone could not distinguish visceral fat mass from subcutaneous fat mass, and the prediction of abdominal subcutaneous fat mass by WC was indeed improved by the addition of BMI as an explanatory factor (39). A retrospective cohort study in Japan recommended a body shape index (ABSI) calculated by dividing WC by an allometric regression of weight and height as an alternative indicator of obesity to replace WC as one of the diagnostic criteria for MetS, since it was more effective in predicting the risk of renal function decline and arterial stiffening (14). In addition, a clear independent relationship between central obesity measured by waist-to-hip ratio (WHR) and albuminuria could be found in the Korean population and non-diabetic South Asians (40, 41). The same goes for waist-to-height ratio (WHtR) (15), both of which are relatively constant anthropometric indexes. Furthermore, visceral adiposity index (VAI), a sex-specific index based on WC, BMI, triglycerides, and HDL-C, has been proved to estimate the visceral adiposity dysfunction associated with cardiometabolic risk (42) and new-onset albuminuria in hypertensive patients (43). Although accumulating evidence indicates that these conventional anthropometric measures have been found correlated with albuminuria in various epidemiological studies, the fact regarding obesity paradox still exists, partly because the apparent correlation among various anthropometric indicators hinders the identification of biologically driven risks for diseases (44). The most other emerging indicators rely on relatively complex empirical mathematical models, which makes it inconvenient to conduct routine examinations among the general population. In a cohort study including 465,629 participants, WWI was proven to be the best predictor of cardiometabolic disease and mortality as compared with BMI, WC, WHtR, and ABSI (45). Consistent with our results, a stronger correlation of WWI and albuminuria compared with BMI and WC was observed in our analysis, suggesting a direct evidence that WWI might be a better predictor for albuminuria than other markers of obesity. Therefore, WWI, as an anthropometric indicator, is expected to be further explored due to its simple calculation and good performance in predicting disease risk.

Albuminuria could be caused by disruption of filter barrier components, including deletion, dedifferentiation and/or loss of podocyte foot process effacement, as well as alterations in glomerular endothelial cells, mesangial cells, and glomerular basement membranes (46). Several potential pathophysiological pathways may support a causal link between abdominal obesity and increased urinary albumin excretion. First, in obesity, higher glomerular capillary pressure (P_*GC*_), renal blood flow, and glomerular filtration rate are noted, thus leading to glomerular damage (47, 48). Second, adipocytes, as an established and active endocrine cell type, can act in paracrine, autocrine, and endocrine ways to secrete various proinflammatory factors, adipokine, and hormones, such as adiponectin and leptin, thus enhancing the inflammatory phenotype of visceral adipocytes in synergy with infiltrating macrophages and ultimately leading to podocyte and endothelial dysfunction. Among them, as a representative, the protective effect of adiponectin on the function and morphology of the renal through regulating inflammation and oxidative stress has been confirmed in many studies (49). On the contrary, its deficiency may lead to increased production of reactive oxygen species, which may even lead to negative effects (50, 51). Third, a status that co-exists with obesity such as expanded plasma volume and hyperinsulinemia also lead to increased renal filtration, affecting renal sodium handling (52, 53).

Our study has several strengths. Our study was based on a nationwide data with sample weights taken into account, so the findings are broadly applicable to the general United States population. Regression analysis was adjusted for covariates, and the large sample size allows us to perform subgroup analyses to confirm the robustness. Finally, we further explored the issue of non-linearity in female participants, thus demonstrating gender differences, which is easily overlooked in everyday clinical practice. However, several limitations also need to be declared. Although we have adjusted some potential covariates, there are still many factors affecting urinary albumin excretion, and we could not completely exclude the influence of other potential confounding factors, such as the use of medications including diuretic, the details of diabetes including its blood glucose control condition and diabetes typing, as well as other social and environmental variables. It was noted that the basic health status and comorbidity of participants, such as hematuria and nephritis including IgA nephritis and glomerulonephritis, may affect data interpretation. However, NHANES did not collect data about this, since we could not further explore our hypothesis with the consideration of hematuria, nephritis, and some other renal disease. In addition, our findings were based on a single country and races, and thus, whether the findings are applicable to other races or countries remains to be investigated. Most importantly, due to the cross-sectional study design, the causality cannot be unraveled.

## Conclusion

This study demonstrated that elevated WWI levels were associated with a higher likelihood of albuminuria, and WWI showed a stronger correlation with albuminuria compared with other obesity markers including BMI and WC, indicating that the management of obesity evaluated by WWI may benefit the kidney health. However, further studies are still needed to validate our findings.

## Data availability statement

Publicly available datasets were analyzed in this study. This data can be found here: www.cdc.gov/nchs/nhanes/.

## Ethics statement

The studies involving human participants were reviewed and approved by the Research Ethics Review Board of the NCHS. The patients/participants provided their written informed consent to participate in this study.

## Author contributions

ZQ: software, data analysis, and writing – original draft. KC: writing – original draft, formal analysis, and methodology. QYa: data analysis. QYu: formal analysis. RL: methodology and funding acquisition. BS: conceptualization, funding acquisition, and writing – reviewing and editing. All authors approved the final version.

## Footnotes

1
http://www.R-project.org

2
www.empowerstats.com
